# Recent Advances on Mutant p53: Unveiling Novel Oncogenic Roles, Degradation Pathways, and Therapeutic Interventions

**DOI:** 10.3390/biom14060649

**Published:** 2024-05-31

**Authors:** Marco Cordani, Alessia Garufi, Rossella Benedetti, Marco Tafani, Michele Aventaggiato, Gabriella D’Orazi, Mara Cirone

**Affiliations:** 1 Department of Biochemistry and Molecular Biology, Faculty of Biological Sciences, Complutense University of Madrid, 28040 Madrid, Spain; 2 Instituto de Investigaciones Sanitarias San Carlos (IdISSC), 28040 Madrid, Spain; 3 Unit of Cellular Networks and Molecular Therapeutic Targets, IRCCS Regina Elena National Cancer Institute, 00144 Rome, Italy; alessia.garufi@ifo.it; 4 Department of Experimental Medicine, University La Sapienza, 00161 Rome, Italy; rossella.benedetti@uniroma1.it (R.B.); marco.tafani@uniroma1.it (M.T.); michele.aventaggiato@uniroma1.it (M.A.); mara.cirone@uniroma1.it (M.C.); 5 Department of Neurosciences, Imaging and Clinical Sciences, University G. D’Annunzio, 00131 Chieti, Italy

**Keywords:** mutant p53, cancer secretome, extracellular vesicles, tumor microenvironment, MDM2, proteasome, ubiquitination, chaperone-mediated autophagy (CMA), heat shock proteins (HSP)

## Abstract

The p53 protein is the master regulator of cellular integrity, primarily due to its tumor-suppressing functions. Approximately half of all human cancers carry mutations in the TP53 gene, which not only abrogate the tumor-suppressive functions but also confer p53 mutant proteins with oncogenic potential. The latter is achieved through so-called gain-of-function (GOF) mutations that promote cancer progression, metastasis, and therapy resistance by deregulating transcriptional networks, signaling pathways, metabolism, immune surveillance, and cellular compositions of the microenvironment. Despite recent progress in understanding the complexity of mutp53 in neoplastic development, the exact mechanisms of how mutp53 contributes to cancer development and how they escape proteasomal and lysosomal degradation remain only partially understood. In this review, we address recent findings in the field of oncogenic functions of mutp53 specifically regarding, but not limited to, its implications in metabolic pathways, the secretome of cancer cells, the cancer microenvironment, and the regulating scenarios of the aberrant proteasomal degradation. By analyzing proteasomal and lysosomal protein degradation, as well as its connection with autophagy, we propose new therapeutical approaches that aim to destabilize mutp53 proteins and deactivate its oncogenic functions, thereby providing a fundamental basis for further investigation and rational treatment approaches for TP53-mutated cancers.

## 1. Introduction

The p53 protein, often referred to as “the guardian of the genome”, plays a crucial role in maintaining cellular integrity by regulating a variety of cellular processes such as DNA repair, cell cycle arrest, apoptosis, and senescence in response to stress and DNA damage [1,2]. As the most frequently mutated gene in human cancers, mutations in *TP53* are present in approximately half of all tumors, leading to the loss of normal p53 function and significantly contributing to cancer development and progression [2,3]. Mutant forms of p53 not only lose their tumor-suppressive capabilities but also gain novel oncogenic functions that actively promote cancer development, metastasis, and therapy resistance. These gain-of-function (GOF) activities of mutant p53 (mutp53) proteins impact various cellular pathways, leading to drastic effects on transcriptional regulation, modulation of signaling pathways, metabolism, immune evasion, and tumor microenvironment [4,5,6,7,8]. The implications of *TP53* mutations extend beyond molecular and cellular effects, influencing clinical outcomes and responses to treatment. Patients with tumors harboring mutant *TP53* often face a poorer prognosis and a more aggressive disease course, underscoring the urgent need for effective therapies targeting the oncogenic functions of mutp53 [9,10].

In this review, we aim to delve into the unique functional and biochemical effects of GOF mechanisms of mutp53, exploring the most recent advances in the literature on how these proteins regulate the intricate network of metabolic pathways and consequently alter cancer secretome and tumor microenvironment (TME), thus leading to the progression of tumor and metastasis. Additionally, we dissect the new and relatively less explored fields of extracellular vesicle regulation by mutp53, which is becoming relevant in the context of cancer associate fibroblasts (CAFs)-cancer cell communication with a drastic impact on disease progression. Furthermore, we will critically examine the degradation pathways that regulate mutp53 levels in cancer cells. Understanding these pathways is crucial for identifying potential therapeutic targets that could mitigate the adverse effects of mutp53 on cancer progression [11]. We will dissect the proteasomal and lysosomal routes of mutp53 degradation, including the role of autophagy in this process. This section will detail how mutp53 evasion of these degradation pathways contributes to its stabilization and accumulation in tumors, highlighting the potential of targeting heat shock protein (HSP) expression and posttranslational modifications to destabilize mutp53 and suppress its oncogenic functions [12,13]. By synthesizing the current understanding and highlighting the gaps in knowledge regarding the degradation pathways involving mutp53, this review underscores the necessity for innovative therapeutic strategies targeting these mutant proteins. While touching upon various cancer types to illustrate the diverse roles of mutp53, our discussion aims to offer insights applicable across a broad spectrum of cancers, hoping to illuminate the complex roles of mutp53 in cancer biology and pave the way for targeted therapeutic interventions. Through this comprehensive exploration, we aspire not only to bridge the current gaps in the understanding of mutp53’s role in cancer but also to inspire future research that could lead to novel and more effective treatments for cancer patients worldwide.

## 2. Novel Mechanisms of Mutant p53 Gain of Function (GOF)

### 2.1. Metabolic Regulations by Mutant p53

**Figure 1:**
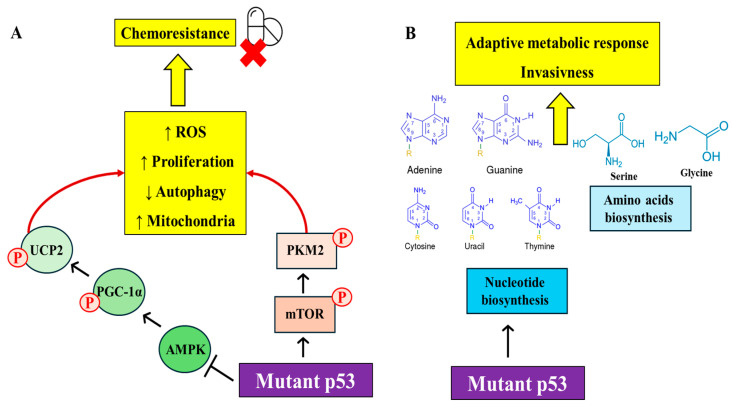
Mutant p53’s oncogenic pathways impacting cellular metabolism and invasion. (**A**) How mutant p53 inhibits the SESN1/AMPK/PGC-1α/UCP2 axis, increasing (↑) ROS levels and cell proliferation while decreasing (↓) autophagy and enhancing (↑) mitochondrial activity, contributing to chemoresistance. (**B**) The adaptive metabolic responses driven by mutant p53, including stimulation of amino acid biosynthesis and nucleotide biosynthesis pathways, fostering cancer cell invasiveness.

Mutp53 exerts complex effects on various metabolic pathways, contributing to cancer cell survival, proliferation, and resistance to therapy. For example, mutp53 isoforms inhibit the SESN1/AMPK/PGC-1α/UCP2 axis, leading to a pro-oxidant state that supports hyper-proliferation, anti-apoptotic effects, and drug resistance in cancer cells. This inhibition suggests the potential of pro-oxidant drugs in treating cancers with mutant *TP53* [14]. Notably, mutp53 also has been shown to induce drastic changes in mitochondrial metabolism, with more aggressive cancer phenotypes demonstrating increased mitochondrial oxidation. This highlights the diversity in metabolic profiles in a mutp53-dependent manner, offering insights for personalized cancer therapy [8]. Mutp53 triggers phosphorylation of PKM2 via mTOR signaling, enhancing aerobic glycolysis, reactive oxygen species (ROS) production, mitochondria activity, and cell proliferation while reducing autophagy, inducing chemoresistance and supporting the oncogenic activity of mutant p53 (Figure 1A). This connection between mutp53 and mTOR stimulation has therapeutic implications, as inhibiting this axis could be effective in cancers carrying mutp53 [15]. In this context, in pancreatic cancer, mutp53 is essential for the formation and maintenance of precursor lesions and alters metabolism to mediate the malignant potential of cancer cells. This alteration includes promoting epithelial–mesenchymal transition (EMT) and invasion, underlining the role of mutp53 role in pancreatic cancer progression [16].

Further expanding on the metabolic influence led by mutp53, recent studies have revealed its role in nucleotide metabolism (Figure 1B). For example, mutp53 can associate with the promoters of nucleotide metabolism genes, enhancing GTP-dependent processes and invasiveness, and creating a dependency on nucleoside salvage pathways. This insight sheds light on the role of mutp53 in driving and sustaining cancer development, correlating with poor prognosis in certain cancers [17]. Additionally, mutp53 has been found to reprogram amino acid metabolism, notably by stimulating serine/glycine synthesis and amino acid intake, crucial for cancer cell survival under nutrient limitations. This represents an adaptive metabolic response dependent on mutp53, highlighting new vulnerabilities in breast cancer treatment [18]. In the context of prostate cancer, the interplay between *TMPRSS2:ERG* fusion and *TP53* mutation has been elucidated, with p53 GOF mutants transactivating genes involved in pyrimidine synthesis, thereby accelerating oncogenesis [19].

### 2.2. Mutant p53 Sustains the Warburg Effect Thus Promoting Lactate Secretion

Lactate, a byproduct of glycolysis, plays a multifaceted role in the tumor microenvironment (TME), influencing tumor response to therapies, and contributing to immune suppression within the TME. Studies show that lactate not only serves as a metabolic precursor and energy source but also modulates immune responses through mechanisms like extracellular acidification and inhibition of the mTOR pathway in immune cells [20].

**Figure 2:**
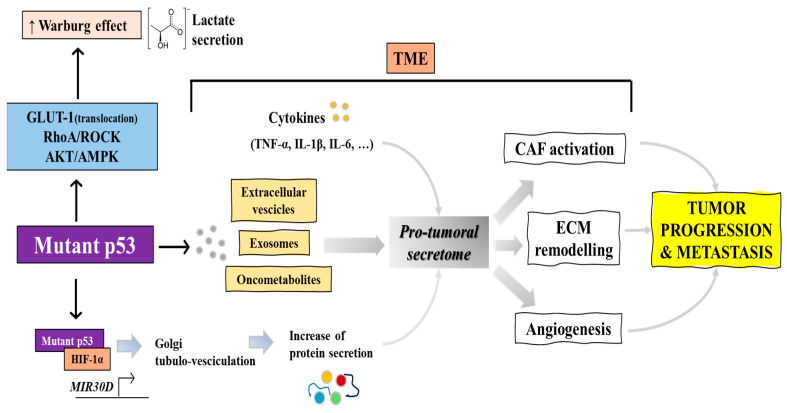
Role of mutant p53 in the enhancement of the Warburg effect and its downstream effects on the tumor microenvironment (TME). Mutant p53 augments glucose uptake through GLUT-1 translocation and stimulates glycolytic signaling pathways including RhoA/ROCK and AKT/AMPK, leading to increased pro-tumoral secretome production. The secretome, enriched with extracellular vesicles, exosomes, and oncometabolites, activates cancer-associated fibroblasts (CAFs), promotes extracellular matrix (ECM) remodeling, and induces angiogenesis, thereby facilitating tumor progression and metastasis. Additionally, mutant p53 interacts with HIF-1α to further support the pro-tumoral secretome and enhance tumor growth and metastatic spread.

Mutp53 has been shown to stimulate the Warburg effect, a mechanism involving enhanced glycolysis and lactate production. This stimulation occurs through mechanisms like promoting GLUT-1 translocation to the plasma membrane and affecting signaling pathways such as RhoA/ROCK and AKT/AMPK, thereby facilitating cancer cell survival and growth [21] (Figure 2). In this context, the work of Eriksson et al. has underscored that the various missense substitutions in TP53, leading to different mutp53 proteins, can distinctly alter cellular metabolism, including both glycolytic and mitochondrial oxidative phosphorylation pathways [22]. This variability in metabolic impact highlights the need for more targeted pharmacological approaches to address the specific variants of mutp53 [22]. Importantly, this metabolic shift not only supports rapid tumor growth but also influences the secretome of cancer cells, impacting the tumor microenvironment and immune evasion [23]. However, the stimulation of the Warburg effect by mutp53 not only enhances lactate production but also sets the stage for broader changes in the cancer cells’ secretome, as discussed below, which represents a direct link between altered metabolism and changes in the tumor microenvironment.

### 2.3. Cancer Secretome Regulation by Mutp53

As anticipated above, mutp53 proteins can significantly modify the cancer cell secretome, producing extracellular vesicles (EVs), exosomes and oncometabolites, thus influencing the tumor microenvironment (TME) with cancer-associated fibroblast (CAF) transdifferentiation, induction of angiogenesis and extracellular matrix (ECM) remodeling, thereby supporting invasive cancer phenotypes (Figure 2). This alteration involves changes in the secretion of enzymes modulating extracellular matrix components, inflammatory cytokines (i.e., IL-6, IL-1β, TNFα), and factors that increase extracellular acidification (Figure 2), crucial for the interaction between cancer and stromal cells [24]. In pancreatic ductal adenocarcinoma (PDAC), where *TP53* is mutated in about 75% of cases, mutp53 isoforms R175H and R273H distinctly affect the cell secretome. This influence extends to cancer progression, including proliferation, chemoresistance, apoptosis, and epithelial–mesenchymal transition (EMT). The discovery of differentially secreted proteins by these p53 mutants, identified through advanced proteomic techniques, points to a potential biomarker signature driven by mutp53 in PDAC [25]. Mutp53 also alters the structure of the Golgi apparatus and function, leading to an increased release of pro-malignant factors in the secretome. This process, mediated through the mut-p53/HIF1α/miR-30d axis, results in enhanced vesicular trafficking and secretion (Figure 2), impacting extracellular matrix remodeling and stromal cell activation within the tumor microenvironment. These changes foster tumor growth and metastatic potential, highlighting the profound effect of mutp53 on the secretory behavior of cancer cells [26].

### 2.4. Mutp53 Regulation of TME and Tumor-Stroma Communication via Extracellular Vesicles (EVs)

Based on the metabolic alterations induced by mutp53, such as the Warburg effect and secretome changes, we now explore its impact on the TME through the regulation of extracellular vesicles (EVs). Cancer-associated fibroblasts (CAFs) in the TME play a pivotal role in cancer progression and immune suppression by remodeling the extracellular matrix (ECM) and secreting signaling molecules [27]. The function of CAFs is intricately linked with EVs, which serve as crucial mediators of intercellular messages in the TME. Extracellular vesicles (EVs), as carriers of molecular information like nucleic acids, proteins, and lipids, are integral to intercellular communication in cancer, influencing steps from tumor development to metastasis [28]. These EVs, laden with molecular signatures, become pivotal mediators of GOF mutp53. In addition to this, mutp53, by altering cellular metabolism, impacts the composition and function of EVs, facilitating the transfer of its invasive properties across the TME. Mutp53 enhances cancer invasiveness by regulating endosomal recycling and exosome production, leading to the transfer of mutp53’s invasive characteristics to other cells and normal fibroblasts. These results in pro-invasive ECM remodeling and potential alteration of distant organ microenvironments [29]. As mutp53 reconfigures the EV landscape, its effects extend beyond mere cell-autonomous changes. Relevant and interesting studies showed that mutp53 proteins can be selectively sorted into EVs, transferring GOF properties to neighboring cancer cells and macrophages, thus promoting tumor growth and creating a supportive TME. This includes the reprogramming of non-cancerous cells and the induction of tumor-supportive cytokines [30]. The p53 R273H mutation alters the tumor microenvironment through exosomal-micro (mi)RNAs, influencing fibroblast activation and cytokines secretion, which, in turn, induce EMT in cancer cells. This highlights a critical circuit involving mutp53-exosomal miRNAs and stromal cell communication [31]. The ramifications of these interactions are profound, illustrating a complex network of mutp53-driven communication within the TME.

Further amplifying its role, the influence of mutp53 permeates through small EVs, affecting a broader range of cells in the TME. In a recent study by Ma et al., it has been shown that GOF p53 protein is packaged into small EVs and transferred to fibroblasts, leading to their transformation into cancer-associated phenotypes and enhancing tumor growth. This transfer is mediated by heat shock protein 90 (HSP90) and reflects the intricate tumor-stromal interactions in cancer [32]. The secretion of mutp53 via exosomes impacts the immune microenvironment, notably by suppressing CD4+ T lymphocytes and altering glycolysis. Targeting this exosome-mediated secretion might offer a therapeutic approach for cancer treatment [33]. In another study, Cooks et al. showed that mutp53 cancer cells shed miR-1246-enriched exosomes, reprogramming macrophages into a tumor-supportive state. This process contributes to an immunosuppressive environment, underlying the role of mutant p53 in involving the immune system to favor cancer progression [34]. The regulatory role of mutp53 in EV production and content not only complements its effects on tumor metabolism but also significantly impacts the TME, highlighting a multifaceted approach of mutp53 in cancer progression.

In this paragraph, we have outlined how mutp53 drives complex metabolic reprogramming, potentially leading to the secretion of various oncometabolites, including lactate and extracellular vesicles (EVs), as well as metabolic sub-products accumulated due to impaired autophagy. These oncometabolites, extending their influence beyond their cells of origin, significantly impact the TME, thereby promoting processes such as tumor invasion, metastasis, and migration (Figure 2). Numerous studies have demonstrated that oncometabolites can alter cellular processes and communication, leading to notable changes in gene expression, signal transduction, and overall cellular behavior, thereby playing a crucial role in cancer progression [35,36]. This highlights that the influence of mutp53 on metabolic pathways might drive cancer progression through the accumulation of oncometabolites, which exert a range of oncogenic effects. These effects span from alterations in cancer signaling pathways and modifications in the TME that favor tumor metastasis to epigenetic changes. Investigating the interaction between mutp53-induced metabolic changes and the dynamics of oncometabolite accumulation offers a promising path for novel therapeutic strategies, particularly those targeting the metabolic vulnerabilities of tumors harboring mutp53. This line of research is vital for advancing personalized cancer treatments.

### 2.5. Other Novel Mechanisms by Which Mutp53 Promotes Cancer Cell Invasion and Metastasis

**Figure 3:**
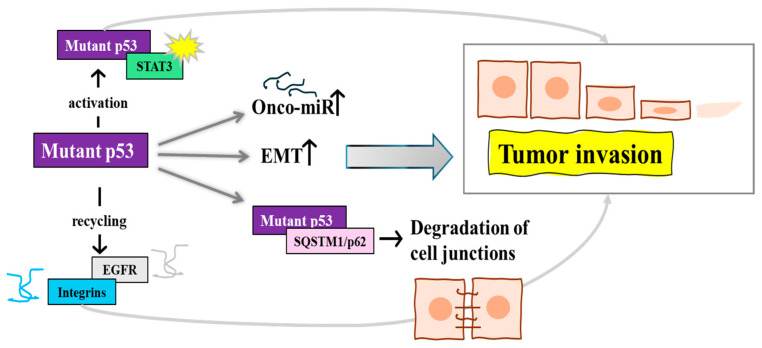
The signaling pathways through which mutant p53 influences tumor invasion. Mutp53 activates STAT3, which is implicated in promoting tumor progression and invasion. Additionally, mutp53 regulates the recycling of EGFR and integrins, integral membrane proteins that are key facilitators of cell migration. Mutp53 also increases Onco-miR production, induces epithelial–mesenchymal transition (EMT) and interacts with SQSTM1/p62, which plays a role in degrading cell-junction-associated proteins, thus enabling cancer cell migration. Collectively, these pathways contribute to enhanced tumor cell invasiveness and the metastatic potential of cancer cells.

Building upon our understanding of the role of mutp53 in altering the cancer cell secretome and remodeling the TME, it is crucial to highlight its direct influence on cellular mechanisms and signaling pathways specifically driving cell migration and invasion. This aspect of the functionality of mutp53 proteins adds another layer of complexity to its oncogenic repertoire, extending beyond its impact on metabolic reprogramming and secretome alterations. For example, Madrigal et al. demonstrated that mutp53 can modulate miRNA expression, such as the upregulation of miR-182-5p, thereby increasing cell migration and invasion by affecting gene regulation pathways linked to these processes [37]. Similarly, in PDAC, colorectal cancers, and glioblastoma mutp53 exhibits GOF activities by binding to and hyperactivating STAT3, leading to enhanced tumor progression, invasion, and metastasis [38,39]. Addressing the influence of mutp53 on cellular trafficking and signaling, recent studies have elucidated how GOF mutp53 impacts endocytic trafficking and signaling of cell surface receptors, crucial for cancer cell motility. Lakoduk et al. showed that the upregulation of Dyn1 by mutp53 enhances the rapid recycling of EGFR and β1 integrins, important for increased cell migration and invasion [40]. Furthermore, the interaction of the p53R273H mutant with proteins like SQSTM1/p62 leads to the degradation of cell-junction-associated proteins, a process that facilitates cancer cell migration [41] (Figure 3). This mutant also affects integrin-mediated tumor-stroma interactions and tumor cell motility by influencing the mutp53-ENTPD5 axis in N-glycoprotein biosynthesis [42]. Moreover, the role of mutp53 in gene suppression is evident in its interaction with tumor suppressor genes. Hotspot mutp53 variants, such as p53-R273H, are known to suppress tumor suppressors like the expression of *KLF6 gene*, impacting E-cadherin levels and promoting cell migration and tumor metastasis, often correlating with poorer survival outcomes in breast cancer [43]. In non-small-cell lung cancer, the p53-R273H mutation upregulates NEU1, a sialidase involved in cell migration, further highlighting the diverse mechanisms through which mutp53 variants enhance cell migration and metastasis [44] (Table 1).

### 2.6. Sirtuin/P53 Axis: Interplay and Potential Speculation

One of the main regulatory mechanisms of p53 transcriptional activity, stability and accumulation in cells is represented by post-translational modifications such as phosphorylation, ubiquitination and acetylation [46]. An increase in the acetylation status of p53 in specific lysine residues stabilizes p53 and promotes transcription and activation of its downstream targets suggesting p53 acetylation as an indispensable post-translational modification that regulates the activity of this tumor suppressor for the proper functioning of the cell [47,48,49]. In previous decades, the discovery of the sirtuin family of proteins has represented an important step for better understanding physiological and pathological processes related to cancer, metabolism, inflammation, etc. Silent information regulator 2 (Sir2) proteins, or sirtuins (SIRTs), are evolutionally conserved enzyme family acting as protein deacetylases/ADP ribosyltransferases [50]. Seven sirtuins have been identified: SIRT1 and SIRT6 are detected in the nucleus while SIRT7 is a nucleolar sirtuin. SIRT2 is a cytoplasmatic sirtuin while SIRT3, SIRT4 and SIRT5 are mitochondrial sirtuins [51]. These proteins were first identified as class III histone deacetylase (HDAC) but their target include also non-histonic proteins, one of them being p53. In particular, p53 is a well-known SIRT1 target of deacetylation and p53-SIRT1 interactions inhibit p53-induced apoptosis and senescence [52,53]. Moreover, the positive feedback loop established between p53 and SIRT1 represses SIRT1 activity maintaining a fine mutual control in physiological conditions [54,55]. While deacetylating and inhibiting p53 activity, SIRT1 also deacetylases Ku70, a DNA repair factor that, once activated, favors the DNA repair process to maintain DNA integrity and stability [56]. For their nuclear localization, SIRT6 and SIRT7 have also been investigated to unravel their putative interaction with p53 but the results so far are contradictory and need further investigations. In recent years, the association between mitochondrial SIRT3 and p53 signaling has been investigated. In their study, Zhang et al. demonstrated that SIRT3 inhibits cell proliferation in hepatocarcinoma cell lines, reducing MDM2-mediated p53 degradation [57]. If the study of the interactions between sirtuins and wild-type p53 is still full of gaps that need to be filled, the study of the interactions between mutp53 and sirtuins is even more lacking [58]. Less is known about the connection between sirtuins and mutp53, but several studies confirm that mutp53 is regulated by acetylation at K382 [59,60]. In their study, Yi et al. demonstrated that YK-3-237, a SIRT1 activator, inhibits the proliferation of Triple-negative breast cancer cell lines by reducing the levels of mutp53 in a SIRT1-dependent manner and enhancing mRNA expression of *PUMA* and *NOXA* with consequent PARP-dependent apoptotic cell death. Furthermore, this small molecule contributes to G2/M arrest accompanied by a decrease in mtp53 [61]. Furthermore, as previously reported, the acetylation status of p53 determines its transcriptional activity that, in turn, is reflected in the transcription of p53-responsive genes [53,62]. In the same way, acetylation affects the activity of mutp53 [63,64]. Tang et al. demonstrated that mutp53 expression levels decreased in small-cell lung cancer cells overexpressing SIRT3 due to increased mutp53 ubiquitination and emphasizing the involvement of SIRT3 in the degradation of mutp53 through the ubiquitin proteasome pathway [65]. Other recent evidence underlines the importance of the SIRT3/mutp53 axis in cancer development. Experiments performed on melanoma cell lines demonstrated that mutp53 induces MnSOD mRNA and protein expression creating a positive regulation of MnSOD activity that, in turn, enhances aerobic glycolysis and results in tumor progression, fueled by a balanced ROS production [45]. MnSOD in fact represents one of the most important targets of SIRT3 in mitochondria where its activity reduces oxidative damage [66]. In their work, Torrens-Mas et al. in fact, demonstrated that mutp53 promotes MnSOD deacetylation activating its enzymatic activity in order to control ROS levels and create a supportive microenvironment for tumor growth. In this context, mutp53 increases SIRT3 and MnSOD activity to maintain ROS levels below a cytotoxic threshold that can enhance cancer development without inducing damage to macromolecules or cellular organelles [45]. Taken together, these data indicate a role of sirtuins in mutp53 regulation that, however, needs further investigation. Since more than 50% of human cancers are characterized by mutations of p53, mutp53 represents a potential target of anti-cancer intervention and Sirtuins could be considered an important tool for mutp53 regulation through its inactivation or degradation. Natural or synthetic compounds with regulatory effects on SIRTs’ activity represent an important approach and a potential therapeutic intervention in cancer development inhibition. Alongside well-known compounds such as resveratrol, honokiol, etc. [67], new synthetic and more specific SIRT activators were synthetized in order to enhance the antitumoral activity of some sirtuins such as mitochondrial SIRT3 [68,69]. Furthermore, new studies revealed that nanostructures such as polyethylene glycol-coated liposomes loaded with flavonoids can modulate sirtuins activity and, in this way, SIRT3/p53 axis or SIRT1 activity [70,71,72]. Therefore, such new sirtuin modulators and promising nanotherapy-based approaches could pave the way toward an effective mutp53 modulation.

## 3. Mechanisms of Mutp53 Degradation

### 3.1. Mutant p53 Degradation: The Proteasomal Route

Differently from wtp53, mutp53 proteins often accumulate to very high levels in human cancers because they may acquire a misfolded and partially denatured conformation with a high tendency to form micro- and macro-aggregates [2] that cannot undergo proteasomal degradation [73]. The hyperstability of mutp53 proteins is at the basis of cancer progression through the gain-of-function (GOF) mechanism. Given the high mutational frequency of p53 in cancer and the GOF activity, mutp53 has become an attractive target for cancer therapies [74]. And mutp53 destabilization is considered a promising therapeutic strategy for cancers carrying mutp53 although, currently, no drugs targeting mutp53 are available for cancer treatment in clinics [75]. Therefore, it is crucial to better understand the mechanisms underlying mutp53 accumulation in order to trigger its degradation.

**Figure 4:**
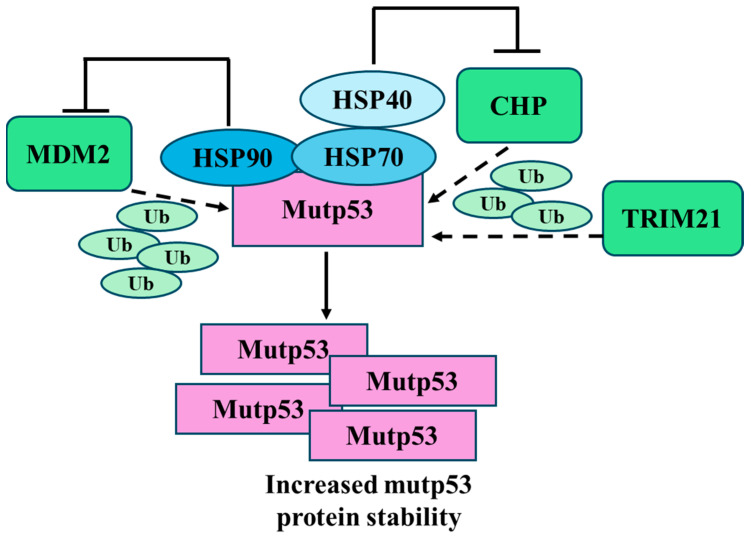
Schematic representation of mutp53 accumulation following impairment of the proteasome system. MDM2 ubiquitinates (Ub, small green symbols) mutp53 for proteasomal degradation, while heat shock proteins HSP90 and HSP70, along with HSP40, stabilize mutp53, preventing its ubiquitination and degradation. HSP90 inhibits MDM2 activity. The co-chaperone protein CHP further ubiquitinates mutp53, enhancing its degradation. HSP40 directly impairs CHIP activity. TRIM21 directly targets mutp53 for ubiquitination and proteasomal degradation, counteracting its accumulation in tumor cells. Impairment of MDM2, CHIP and TRIM activity leads to increased mutp53 stability.

Several mechanisms such as those proteasome- or autophagy-mediated have been shown to trigger mutp53 degradation. It is well known that the proteasome and macroautophagy (herein as autophagy) regulate the half-life of many proteins [76] including wild-type p53 [77]. While the proteasome predominantly targets short-lived ubiquitinated proteins, autophagy mainly degrades long-lived misfolded proteins, intracellular aggregates or damaged organelles. In addition, cross-talk between autophagy and the ubiquitin proteasome system (UPS) exists, as blockage of one pathway can lead to activation of the other [78]. One of the mechanisms that induce mutp53 stabilization is the inhibition of MDM2 ubiquitin ligase activity. Studies from genetically engineered mouse models (i.e., R172H mutp53-knockin mice), together with in vitro studies, demonstrated that MDM2 can effectively degrade mutp53 in normal tissues but not in tumor tissues [79,80]. Furthermore, MDM2 deletion of R172H mutp53 in knock-in mice results in mutp53 accumulation in normal tissues, which in turn promotes tumor development and reduced mouse life span [81]. Tumors develop specific mechanisms to impair MDM2-mediated mutp53 degradation, leading to mutp53 accumulation [81,82,83,84]. For instance, components of the Hsp90 chaperone machinery, a stress-induced system that supports cancer cell survival by counteracting protein misfolding and toxic aggregation [85], bind to mutp53 and inhibit MDM2 ubiquitin-protein isopeptidase ligase function by concealing the ARF-binding site on MDM2, leading to mutp53 accumulation [86]. The system includes Hsp90, Hsp70 and other co-chaperones such as Hsp40/DNAJA1. Both Hsp40 and CHIP interact with Hsp70, which cooperates with HSP90 in stabilizing mutp53 [13]. Thus, Hsp40/DNAJA1 inactivates CHIP, protecting mutp53 from CHIP-mediated degradation [87] (Figure 4). This mechanism can be disrupted by statins, cholesterol-lowering drugs, that, by reducing the level of mevalonate-5-phosphate in the mevalonate pathway (MVP), induce CHIP-mediated degradation of mutp53 [87]. Accordingly, c-Myc inhibition has been shown to reduce the expression of mevalonate kinase (MVK), a molecule belonging to the mevalonate pathway, and reduce mutp53 stability [88].

Recently, a new mechanism for mutp53 degradation via proteasome has been uncovered. The authors, by screening for specific mutp53-interacting proteins, identified human tripartite motif 21 (TRIM21) as a critical E3 ubiquitin ligase of mutp53 [89] (Figure 4). They found that TRIM21 directly interacts with mutp53 but not wild-type p53 and that TRIM21 deficiency in cancer cells promotes mutp53 accumulation and GOF in tumorigenesis. These results are supported by the finding that TRIM21 is frequently downregulated in some human cancers, including colorectal and breast cancers, and low TRIM21 expression is associated with poor prognosis in patients with cancers carrying mutp53 [89].

### 3.2. Mutant p53 Degradation: The Lysosomal Routes

**Figure 5:**
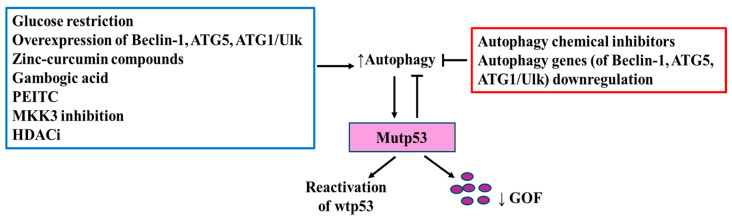
Mutant p53 degradation by autophagy. Activation (↑) of autophagy by different conditions (summarized in the blue rectangle) induces mutp53 degradation (purple small circles) leading to reduction (↓) of GOF; sometimes, degradation of mutp53 reactivates the wild-type function. Inhibition of autophagy by different mechanisms (summarized in the red rectangle) impairs mutp53 degradation.

Beyond macroautophagy (herein as autophagy), micro- (MI) and chaperone-mediated autophagy (CMA) pathways, are key components of the cellular machinery that play important roles in lysosome-mediated protein degradation [90]. Autophagy is a multistep process that involves the sequential formation of a double-membrane structure, the phagophore, that ultimately fuses with lysosomes to degrade sequestered cargos via the activity of hydrolases in autolysosomes [91]; MI involves the direct uptake of cargo material by the lysosomal or vacuolar membrane [92]; CMA selects proteins with a pentapeptide motif related to KFERQ that is recognized by the heat shock cognate 71 kDa protein (Hsc70 (also known as HSPA8)) and co-chaperones, forming a chaperone complex that enables the translocation of the cargo protein into the lysosomal lumen via binding the lysosomal receptor, lysosome-associated membrane protein 2A (LAMP-2A) [93,94]. Certain mutp53 proteins have been shown to be degraded through autophagy but also to regulate autophagy [90]. For instance, glucose restriction in multiple cancer types, bearing the p53R175H, R280K mutants, was shown to induce p53 mutant deacetylation, routing it for degradation via autophagy [64,95]. In agreement, inhibition of autophagy with chemical inhibitors or by downregulation of the essential autophagic genes ATG1/Ulk1, Beclin-1 or ATG5, results in mutp53R175H and R280K stabilization, conversely, overexpression of Beclin-1 or ATG1/Ulk1 leads to p53 mutant depletion [11] (Figure 5). In order to trigger mutp53 degradation through autophagy, several classes of small molecules that promote this catabolic process have been tested. Some of them include the curcumin-based zinc compound (Zn(II), curcumin compounds and capsaicin (8-methyl-N-vanillyl-6-noneamide), which have been shown to deplete the expression of p53RH175 and p53R273H mutants [96,97,98], Gambogic acid, a pro-apoptotic molecule that promotes the autophagy-mediated p53R280K and p53S241F degradation [99], inhibition of MKK3, a dual protein MAP kinase, which reduces p53R273H mutant protein levels through ER stress-induced autophagy [100], the cruciferous-vegetable-derived phenethyl isothiocyanate (PEITC), which triggers p53R175H, R273H, R248Q mutant degradation [101], and histone deacetylases inhibitors (HDACi), which stimulate autophagy and degrade p53R172H, R248Q, R280K mutants [102]. Degradation of mutp53 proteins leads to the reduction of GOF; however, sometimes it also reactivates the wild-type p53 function, restoring cancer cell sensitivity to cytotoxic agents [96,103,104] (Figure 5).

**Figure 6:**
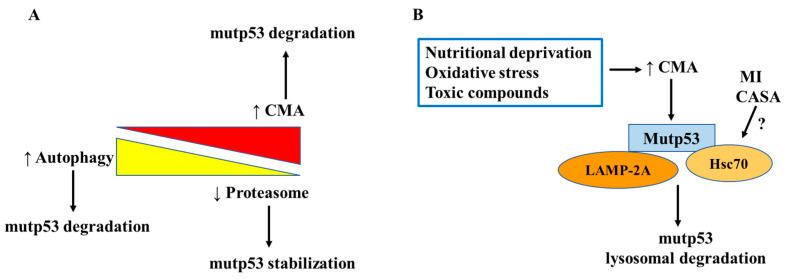
Degradation pathways for mutant p53. (**A**) Schematic representation of the balance between the different degradative routes and the stabilization/degradation of mutp53. Blocking (↓) of the proteasome upregulates (↑) autophagy while reduction of autophagy activates (↑) chaperone-mediated autophagy (CMA). (**B**) Activation (↑) of CMA by different conditions (in the blue rectangle), triggers mutp53 binding to both Hsc70 and LAMP-2A, chaperoning mutp53 to the lysosomes for degradation. The potential effect (?) of mutp53 degradation by microautophagy (MI) or chaperone-assisted selective autophagy (CASA) through Hsc70 is also shown.

Cells respond to blockage of the proteasome by up-regulating autophagy, whereas inhibition of autophagy under nutritional deprivation conditions has been shown to activate chaperone-mediated autophagy (CMA) [94] (Figure 6A). Thus, CMA is maximally activated in response to stressors such as prolonged starvation, exposure to toxic compounds, or oxidative stress [105]. In the study by Vakifahmetoglu-Norberg et al., the authors show that suppression of macroautophagy, in confluent cells, promoted the degradation of mutant p53 through CMA. The authors demonstrate that mutant but not wild-type p53 bound both Hsc70 and LAMP-2A, chaperoning mutp53 to the lysosomes [106] (Figure 6B). Several mutant p53 proteins, including P98S, P151H, A161T, R175C, R175D, R175H, L194F, S227K, S227R, G245C, R248L, R248W, E258K, R273H, R273L, R280K, and R28W, have been shown to be targeted by CMA in cancer cells and in in vitro experiments with ectopic overexpression of p53 mutants [106,107]. We have recently shown that ER stress activation induced CMA which contributed to lysosomal mutp53 degradation [108]. The findings suggest that activating CMA-mediated mutant p53 degradation may be more efficacious than treatment with targeted mutant p53 specific reactivating small molecules, along with the fact that the activation of CMA was not or less effective on wild-type or p53 null expressing cancer cells [106].

High LAMP-2A levels have been shown to correlate with a predisposition of CMA, whereas silencing of LAMP-2A blocks the degradation of proteins via the CMA pathway [109]. Transcriptional control of LAMP-2A expression was shown to be under the control of the NFE2L2/NRF2 (nuclear factor erythroid 2-related factor 2 (NRF2) [110], the master regulator of the oxidative response. Interestingly, NRF2 has been shown to interact with mutp53R280K contributing to selective activation of NRF2 downstream transcriptional program [111]. This cross-talk suggests that mutant p53 proteins might contribute to CMA activation through NRF2-mediated LAMP-2A transactivation. However, to date, the role of CMA in tumorigenic conditions is not well defined and there are no direct pharmacological CMA activators for cancer cells.

Other mechanisms that trigger protein degradation are microautophagy (MI), a process that induces lysosome-dependent protein degradation following recognition of proteins harboring a KFERQ-like motif by the molecular chaperone HSC70 [112], and chaperone-assisted lysosomal degradation pathway CASA (chaperone-assisted selective autophagy), which also requires the involvement of HSC70. Since the amino acid sequence of p53 contains KFERQ-like motifs that are recognizable by HSC70, it is plausible that the mutant p53 protein might be targeted by MI or CASA (Figure 6B). However, it is currently not known whether wild-type and/or mutp53 proteins are targeted and degraded by these pathways. At the moment, it is also unclear if the distinct tendency of mutp53 proteins to misfold or aggregate may affect the susceptibility for recognition by the different degradation pathways and targetability. Another important aspect to be considered is whether endoplasmic reticulum (ER) stress and UPR activation can affect mutp53 degradation in cancer cells that display a high level of basal stress, considering that ER stress is strongly interconnected with the activation of autophagy and/or CMA [113]. A recent study showed that in conditions in which autophagy is activated during ER stress the degradation of mutp53 mainly occurs through this route and Ire1 alpha UPR sensors are involved in such an effect [114].

### 3.3. Autophagy Regulation by Mutant p53 and Its Impact on TME

Autophagy, a self-degradative process crucial for maintaining cellular homeostasis, plays a complex dual role in cancer [115]. Autophagy aids in both the survival of tumor cells under stress and acts as a tumor suppressor through the elimination of damaged organelles and proteins. The intricate involvement of autophagy in cancer underscores its importance in both tumor progression and suppression [116]. Notably, autophagy is closely intertwined with TME, particularly in metastasis and EMT. It helps tumor cells adapt to microenvironmental changes and avoid anoikis, enhancing their invasive capabilities [117,118]. In melanoma, for instance, the deletion of the autophagy-related gene Ambra1 leads to accelerated tumor growth and increased metastasis [119]. It is well established that mutp53 proteins can exert their oncogenic functions by inhibiting cell death autophagy, thus promoting cancer cell proliferation and resistance to apoptosis. This can be achieved through the suppression of key autophagy-related proteins and pathways, such as BECN1, DRAM1, ATG12, and AMPK, while stimulating the mTOR signaling pathway [120]. In this frame of studies, mutp53 has been shown to bind directly to the AMPKα subunit, inhibiting AMPK activation and consequently autophagy, further enhancing the oncogenic potential of mutp53 [121]. The localization of p53 mutants in the cytoplasm plays a crucial role in autophagy inhibition. Indeed, certain p53 mutants that localize predominantly to the cytoplasm have been found to effectively repress autophagy, further linking the cellular distribution of p53 to its impact on autophagic processes and cancer progression [122].

### 3.4. Targeting HSP Expression and Posttranslational Modifications to Destabilize Mutp53

It is now quite evident that the aberrant accumulation of mutp53 represents an Achille’s hill of cancer cells addicted to mutp53 high expression levels and thus reducing its stability represents a promising avenue from the therapeutic point of view. Also considering that there are few possibilities to directly inhibit the oncogenic functions of mutp53, as reported for other “undruggable” oncogenes [123,124]. The reduction in HSP expression as well as the inhibition of their capacity to interact and stabilize mutp53 may be a strategy to destabilize mutantp53 proteins.

HSPs, classified into six families based on molecular weight, include small HSPs (e.g., HSP27), HSP40, HSP60, HSP70, HSP90, and HSP110. Each family comprises several members with different subcellular localizations and functions [125]. Among those, the most studied in the context of mutp53 are HSP90 and HSP70, which exist in diverse isoforms, differently involved in the folding, stabilization, and degradation of numerous client proteins, including mutp53 [126]. Whereas wtp53 transiently interacts with HSPs, several mutant p53 proteins have been shown to stably associate with those molecules, being one of the underlying mechanisms leading to mutp53 accumulation [127]. The dependency of mutp53 on HSPs has been acknowledged since 1996 [128], when it was shown that the binding of HSP90 to the DNA binding domain (DBD) of mutp53 inhibits E3 ligases MDM2 and CHIP, preventing mutp53 proteasomal degradation [128]. Mutp53 GOF strongly relies on stabilization mediated by the Hsp90/Hsp70/Hsp40 chaperone mechanism, which protects mutant p53 from being degraded by ubiquitination of MDM2 and other E3 ligands [13,129] (Figure 4). Interestingly, as often observed in nature, a feedback loop regulates the equilibrium between HSP90 and mutp53. Indeed, if HSP90 prevents mutp53 degradation, the latter gives back the favor to HSP90, activating heat shock factor 1 (HSF1), the main transcription factor regulating HSP expression, via enhanced receptor tyrosine kinase (Her2, EGFR) signaling, increasing the expression of HSP90 [130] (Figure 6). As mutp53 can activate HSF1, this effect results in the transcription not only of HSP90 but also of other HSPs, including HSP70 and HSP27, whose hyperexpression, as for HSP90, is detected in most cancers. Regarding HSP70, and in particular its inducible form HSP72, it has been reported that it contributes to mutp53 stabilization, not only by cooperating with HSP90 [126] but also with the co-chaperones BAG1 and HSP40 [131] (Figure 6). The positive feed-forward loop with HSP90 has been reported for either conformational or DNA contact mutant p53 proteins (i.e., R273H, R175H, R280K) that, through this interplay, reinforce their oncogenic signaling. It has been recently reported that the cross-talk between mutp53 and HSP90 sustains the interplay occurring between two other molecules involved in cancer survival, NRF2 and p62/SQSTM1, with important implications on the anti-oxidant response and response to therapies [132,133].

**Figure 7:**
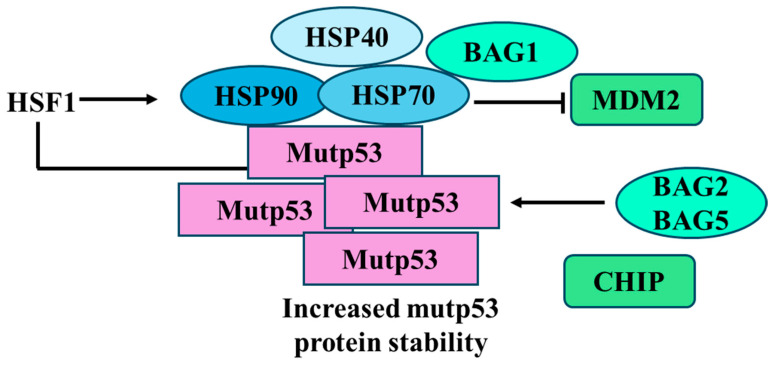
Interaction of mutant p53 with molecular chaperones and co-chaperones that regulate its stability within the cell. Heat shock proteins HSP90 and HSP70, along with co-chaperone HSP40 and BAG family members BAG1, BAG2, and BAG5, are depicted binding to mutp53 to stabilize and prevent its degradation. Mutp53 contributes to increasing the HSP90 expression through the HSF1 transcription factor. On the other hand, HSP70 blocks the MDM2 function and BAG2 and 5 block the CHIP activity. These interactions contribute to increased mutp53 protein stability.

Furthermore, it has been reported that HSP70, (a term by which we will hereafter refer to the inducible form HSP72), forms aggregates with MDM2, inhibiting MDM2-mediated ubiquitination and degradation of mutp53 (R175H) and promoting its aggregation and stabilization [13] (Figure 7). Romeo et al. recently investigated the role of HSP70 in pancreatic cancer cells carrying different p53 mutations and showed that the HDAC inhibitors Valproic Acid (VPA) and Tricostatin (TSA) downregulate HSP70 and through this mechanism reduced mutp53 expression level [134]. However, the role of HSP70 remains controversial, as another study has shown that a compound capable of increasing the interaction between HSP70 and mutp53 restores the wtp53 conformation in mutp53-bearing hepatocarcinoma cells [135], similar to what has been reported for small molecules that activate Hsp40 [136]. Hsp70 and DNAJB1 (another member of the HSP40 family) may also increase the local unfolding of both wild-type and mutant p53-DNA binding domain, an effect counteracted by HSP90 [126]. Regarding co-chaperones of the BAG family, besides cooperating with HSPs, as in the case of BAG1, other members, such as BAG2 and BAG5, have been reported to directly sustain mutp53 expression by binding to it and preventing the interaction with CHIP and MDM2 [75] (Figure 6). These findings suggest that the interplay between HSP90, HSP70, BAGs, HSP40 and mutp53 is far from being fully understood. Even more controversial is the role of HSC70, the constitutive form HSP70, also named HSP73. Indeed, if on the one hand, Hsc70, together with Hsp40, Hop and ATP, contributes to forming a stable complex with Hsp90 and with the conformational mutant p53R175H [137], on the other hand, HSC70/HSP90-association with this mutp53 may promote the binding to CHIP and target mutp53 for proteasomal degradation [138]. Accordingly, previous studies have shown that by binding to the carboxyl termini of Hsc70 and Hsp90, CHIP induces the ubiquitination of other chaperone-bound client proteins and together with Ubc4/5 family E2 enzymes promotes the degradation of client proteins via the proteasome [139]. Last but not least, binding HSC70 to mutp53 can bring it to lysosomes to be degraded via CMA, as mentioned in the previous paragraph [106]. Regarding HSP40, in particular the DNAJA1 isoform, in addition to behaving as a co-chaperone with HSP90, supporting the stabilization of mutp53, it can prevent proteasomal degradation of misfolded or conformational mutp53 and DNAJA1 inhibition induces mutp53 folding and wtp53 reactivation [140]. Recently, a new molecule, compound **7-3**, was identified which, by reducing the expression level of DNAJA1, depleted conformational mutp53 [141], further highlighting the key role of this co-chaperone in the stabilization of mutp53.

**Figure 8:**
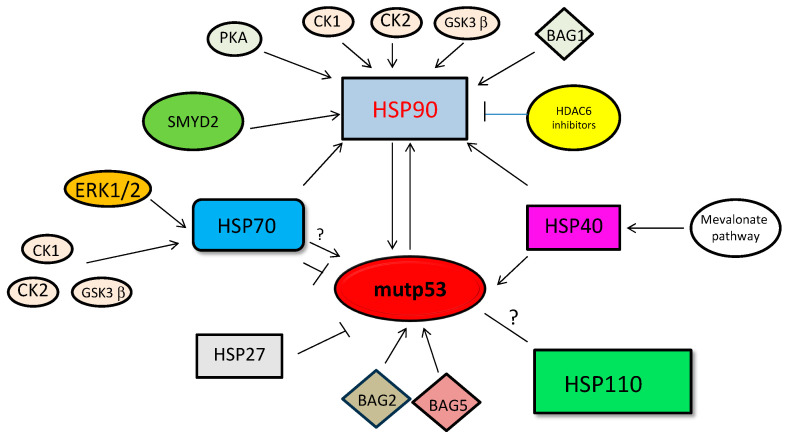
Schematic representation of HSPs inactivation/activation. HSPs regulation by small molecules or by post-translational modifications.

Regarding the small HSP27, whose hyperexpression and role in cancer cell survival has been largely demonstrated [142], it is known to interact with wtp53 [143] but if it could regulate mutp53 expression is not completely clarified, although its overexpression seems to deplete mutp53 rather than stabilizing it [144]. In a recent study it was shown that, although downregulated by a c-Myc inhibitor, HSP27 did not contribute to the reduction of mutp53 expression level induced by such a treatment [88]. Even less known is the role of HSP110 in the regulation of mutp53 stability, also because no specific inhibitors for this HSP are yet commercially available. However, it is important to note that in addition to employing small molecules that directly inhibit HSPs, such as 2-phenylethynesulfonamide (PES) for HSP70, geldanamycin for HSP90, or compound **7-3** for HSP40, we can also alter the activity of HSPs by inducing post-translational modifications (PTMs) of these proteins. This represents a promising strategy to interfere with the capacity of HSPs to regulate mutp53 stability. Given the complex pattern of PTMs and the importance that they play in regulating HSP functions, the term “chaperone code” has been created for some HSPs such as HSP70 and HSP90 [145,146]. For example, phosphorylation of HSP70 mediated by ERK1/2 has been shown to increase its folding capacity [147]. Furthermore, C-terminal phosphorylation of Hsp70 as well of Hsp90, by CK1, CK2 and GSK3-β, favor the binding to HOP while preventing that to CHIP, facilitating the protein folding over protein ubiquitin-mediated degradation, two opposite functions that these HSPs may play [148]. Also, the phosphorylation of HSP90 by PKA may positively influence its interaction with mutp53 [149] (Figure 8). Considering that HSP70 and HSP90 can be phosphorylated at different residues, the impact of this PTM on their function remains still incomplete and the same also applies to the kinases and phosphatases that regulate HSP phosphorylation.

Among PTMs, acetylation and methylation have also been reported to regulate HSP activity, and interestingly, these PTMs could influence or be influenced by their phosphorylation changes. Therefore, understanding how to manipulate PTMs may offer a promising opportunity to compromise the ability of HSPs to stabilize oncogenic molecules, including mutp53. However, it is not easy to understand how to carry this out, since, for example, the intensity of HSP70 acetylation, mediated by the acetyltransferase ARD1, appears to vary in a time-dependent manner in stressed cells, resulting in a different outcome on regulating its binding to the co-chaperones CHIP and HOP, which regulate the balance between folding and degradation of HSP70 client proteins [150]. Furthermore, as mentioned above for phosphorylation, acetylation can have a different impact on HSP70 function, depending on the lysine undergoing acetylation, the cellular context, and the agents that mediate acetylation/deacetylation [145,150].

Studying the impact of HSP acetylation on the stability of mutp53 has mainly focused on HSP90 [133], which can be acetylated on several residues [133]. Acetylation of HSP90 by the HDAC6 inhibitor vorinostat (SAHA) has been shown to impair its ability to stabilize mutp53, highlighting that the deacetylated state is critical for HSP90 function [151,152] (Figure 8). Furthermore, FK228, a class I HDAC inhibitor, has been reported to impair the stabilizing capacity of HSP90 as it induces the acetylation of Hsp70 and reduces cooperation between the two HSPs [153]. Given the importance of HSP70/HSP90 cooperation in the stabilization of mutp53, it is plausible to hypothesize that FK228 could reduce mutp53 expression. In addition to acetylation, farnesylation may regulate HSP function, as in the case of HSP40/DNAJA1 which, following this PTM may more efficiently sustain mutant p53 stability and oncogenesis [154]. Indeed, the mevalonate pathway playing a key role in inducing such posttranslational modification can block E3 ubiquitin ligase CHIP and its-mediated degradation of mutant p53 (Figure 8) [87]. The methylated or nonmethylated state can also influence the chaperoning activity of HSP70 and HSP90. Indeed, when dimethylated by SETD1A, HSP70 changes its localization from cytoplasmic to nuclear [155] while HSP90 methylation induced by SMYD2 promotes its dimerization and chaperone complex formation (Figure 8) [156]. Investigating the impact of HSP methylation on mutp53 stabilization may unveil new strategies to interrupt the criminal alliance between these proteins. Moreover, it should be considered that the expression of HSP may be influenced by DNA methylation of their promoters or other genomic regions as well as by methylation of histones, because the latter may also undergo acetylation, sometimes at the same lysine residues [157]. This suggests that epigenetic changes may strongly affect the expression and function of HSPs and therefore may have a crucial influence on mutp53 expression, holding great promise in the treatment of cancers carrying p53 mutations. However, to make this complicated story even more complicated, HSPs can themselves induce epigenetic changes, for example, HSP90 has been shown to strongly influence histone methylation [158].

## 4. Conclusions and Perspectives

In summary, this work extensively explored the contribution of mutp53 to cancer cell invasion and metastasis and its central role in the disease’s pathogenesis. Based on our thorough review of the literature, the functions of the mutp53 protein are not limited to the DNA damage response and cell cycle control. Furthermore, the interaction of mutp53 with sirtuins is an interesting new aspect of p53 biology that affects the oncogenic role and stability/degradation of this protein. The recent literature has revealed the deep involvement of GOF mutp53 in various signaling pathways, significantly increasing the aggressiveness, mobility, and invasiveness of cancer cells. Additionally, cooperation with metabolic reprogramming, the cancer secretome, and mutual interdependence with the TME has established an oncogenic network that not only nourishes the growth of a malignant tumor but also becomes a niche in which malignant cells grow, survive, and avoid treatment. Hence, the emerging roles of mutp53 in modulating aspects of cancer biology demonstrate a molecular promiscuity that transforms a genomic guardian into a promoter of malignancy. The altered network of pathways suggests that mutp53 acts as a master regulator within the adaptive landscape of tumor cells, creating new opportunities for targeted therapeutic interventions. In this context, the insights from recent studies provide a solid foundation for creating new drugs that allow selective degradation of mutp53 or suppression of its oncogenic activity, which ultimately may restore normal p53 tumor-suppressive function and increase cancer cell sensitivity to treatment (Table 2).

Expanding upon the therapeutic targeting of mutp53 presents a compelling target for therapeutic intervention. This interaction holds the potential not only to restore the tumor-suppressive function of p53 but also to facilitate the degradation of its mutant forms, which could be pivotal in re-sensitizing cancer cells to treatment. Precision in discriminating between mutant and wild-type forms of p53 is crucial. This precision relies on a deep understanding of mutp53’s unique conformational structures and their specific binding affinities. The advent of sophisticated screening methods and structure-based drug design holds promise for the creation of novel therapeutics, such as allosteric modulators, that could either restore wild-type p53 function or specifically target mutp53 for degradation. Advances in understanding the degradation pathways for mutp53, including autophagy, the proteasome, and lysosome-associated routes, have revealed new therapeutic avenues. Our review spotlights the intricate roles of chaperones like HSP90 and co-chaperones such as BAG proteins in stabilizing and folding mutp53, suggesting that targeting these interactions with inhibitors could undermine mutp53’s stability, facilitating its degradation. Likewise, bolstering the ubiquitin-proteasome system to efficiently identify and dismantle mutp53 through the manipulation of E3 ligases and deubiquitinating enzymes is an enticing approach. Additionally, the evolving narrative of autophagy’s reciprocal regulation by mutp53 introduces both complexity and potential. The consideration of autophagy inducers, which may clear mutp53 aggregates and re-sensitize resistant cancer cells to chemotherapy, is rapidly gaining scientific traction. Moreover, targeting the metabolic susceptibilities engendered by mutp53 might yield combination metabolic therapies that selectively impair cancer cells harboring this mutant protein. The future of mutp53-targeted therapies could be revolutionized by exploiting computational biology, capable of modeling mutp53 interaction networks [159], with experimental pharmacology. Insights into the post-translational modifications that govern chaperone interactions with mutp53 open up innovative avenues to influence these complex molecular relationships. These advancements pave the way toward a future where targeting mutp53, once a daunting challenge, becomes a pivotal element in personalized cancer therapy development.

## Figures and Tables

**Table 1:** Impact of distinct GOF p53 mutations in cancer: Roles, targets, and mechanisms.

Mutant p53 Proteins	Role in Cancer	Biological Effect Led by Mutant p53	References
p53R175H, p53R273H	Induces changes enhancing cancer aggressiveness	Triggers metabolic alterations increasing mitochondrial oxidation	[8]
p53R175H, p53R273H	Enhances pro-oxidant state and drug resistance	Inhibits the axis leading to hyper-proliferation, anti-apoptotic effects, and drug resistance	[14]
p53R273H, p53R280K	Enhances glycolysis and ROS production	Phosphorylation of PKM2 via mTOR signaling, reduces autophagy, supports oncogenic activity	[15]
p53R273H	Alters metabolism promoting EMT and invasion	Essential for precursor lesions, mediates malignant potential through metabolic changes	[16]
p53R248Q, p53R273H	Enhances invasiveness and cancer development	Associates with promoters of nucleotide metabolism genes, enhances GTP-dependent processes	[17]
p53R248Q, p53R273H	Stimulates synthesis crucial for survival under nutrient limitations	Reprograms metabolism, particularly serine/glycine synthesis, crucial in breast cancer	[18]
p53R273H, p53R280K	Accelerates oncogenesis	Transactivates genes involved in pyrimidine synthesis, associated with TMPRSS2-ERG fusion in prostate cancer	[19]
p53R175H, p53R248Q, p53R273H	Stimulates enhanced glycolysis and lactate production	Promotes GLUT-1 translocation, affects RhoA/ROCK and AKT/AMPK signaling, enhances cancer cell survival and growth	[21]
p53R175H, p53R248Q, p53R273H	Alters cellular metabolism	Influences both glycolytic and mitochondrial oxidative phosphorylation pathways, impacts metabolic diversity	[22]
p53R175H, p53R248Q, p53R273H	Influences the secretome impacting immune responses	Shift in metabolism influences the secretome of cancer cells, impacting tumor microenvironment and immune evasion	[23]
p53R175H, p53R273H	Supports invasive cancer phenotypes	Changes in secretion of enzymes, inflammatory cytokines (IL-6, IL-1β, TNFα), and factors increasing extracellular acidification	[24]
p53R175H, p53R273H	Affects cancer progression	Alters cell secretome impacting proliferation, chemoresistance, apoptosis, EMT	[25]
p53R175H, p53R273H	Enhances malignant factor release	Alters Golgi structure and function, mediated by mut-p53/HIF1α/miR-30d axis leading to increased pro-malignant factor release	[26]
p53R273H, others not specified	Enhances cancer invasiveness	Alters the composition and function of EVs, facilitating the transfer of invasive properties across the TME.	[29]
p53R273H	Modifies TME, supports tumor progression	Transferring gain-of-function properties to neighboring cells and macrophages, thus promoting tumor growth	[30]
p53R273H	Influences ECM remodeling and EMT	Alters the TME through exosomal-micro (mi)RNAs that impact fibroblast activation and cytokine secretion, promoting EMT and enhancing tumor-stroma communication.	[31]
p53Y234C, p53G245D, p53I195T, p53R248W, p53R273H, p53Y163C	Leads to cancer-associated phenotypes and enhances tumor growth	GOF p53 proteins are packaged into small EVs and transferred to fibroblasts, mediated by HSP90, activating pathways like Nrf2 that induce cancer-associated transformations	[32]
p53R273H	Suppresses immune responses, notably CD4+ T lymphocytes, and alters glycolysis	Mutp53 secretion via exosomes impacts the immune microenvironment	[33]
p53R273H, p53R249S	Reprograms macrophages into a tumor-supportive state	Cancer cells shed miR-1246-enriched exosomes, which reprogram macrophages, contributing to an immunosuppressive environment	[34]
General mutp53 (specific mutations not detailed)	Enhances cell migration and invasion	Modulates miRNA expression, such as upregulation of miR-182-5p, affecting gene regulation pathways related to migration and invasion	[37]
General mutp53 (in PDAC, colorectal cancers, glioblastoma)	Promotes tumor progression and metastasis	Binds to and hyperactivates STAT3, leading to enhanced tumor progression, invasion, and metastasis	[38,39]
p53R273H	Increases cell migration and invasion	Enhances rapid recycling of EGFR and β1 integrins, crucial for cancer cell motility	[40]
p53R273H	Facilitates cancer cell migration	Interacts with SQSTM1/p62 leading to the degradation of cell-junction-associated proteins	[41]
p53R273H, p53R248W, p53R248Q, p53R280K	Affects tumor cell motility	Influences the mutp53-ENTPD5 axis in N-glycoprotein biosynthesis, impacting tumor-stroma interactions and motility	[42]
p53R273H	Promotes cell migration and metastasis	Suppresses tumor suppressor genes like KLF6, impacting E-cadherin levels and promoting cell migration and metastasis	[43]
p53R273H	Enhances cell migration	Upregulates NEU1, a sialidase involved in cell migration, highlighting diverse mechanisms enhancing metastasis	[44]
p53R273H, p53E258K	Enhances tumor progression	Mutp53 induces MnSOD mRNA and protein expression, enhancing aerobic glycolysis and tumor progression through balanced ROS production and reduced oxidative damage	[45]

**Table 2:** Treatments leading to mutp53 degradation.

Mutant p53 Proteins	Agents/Compounds	References
p53R175H, p53R273H	curcumin-based zinc compound (Zn(II), curcumin compounds and capsaicin (8-methyl-N-vanillyl-6-noneamide)	[96,97,98]
p53R280K, p53S241F	Gambogic acid	[99]
p53R273H	inhibition of MKK3 reduces mutp53 levels through ER stress-induced autophagy	[100]
p53R175H, R273H, R248Q	cruciferous-vegetable-derived phenethyl isothiocyanate (PEITC),	[101]
p53R172H, R248Q, R280K	histone deacetylases inhibitors (HDACi)	[102]
P98S, P151H, A161T, R175C, R175D, R175H, L194F, S227K, S227R, G245C, R248L, R248W, E258K, R273H, R273L, R280K, R28W	Stimulation of CMA by macroautophagy inhibition or by inducing ER stress	[106,107,108]
R273H, R175H, R280K	HSP90 inhibition	[129]
p53R280K, p53R273K, p53L194F, p53R175H, p53P223L	HSP90, HSP70 acetylation	[152,153]
p53 R280K, p53 R273H, p53 M237I, p53 R249S, p53 R175H, p53 L194F p53 V157F, p53 R249S p53 R213Q	Mevalonate pathway inhibitors	[87]

## References

[1] Levine A.J., Momand J., Finlay C.A. (1991). The p53 tumour suppressor gene. Nature.

[2] Muller P.A.J., Vousden K.H. (2013). p53 mutations in cancer. Nat. Cell Biol..

[3] Greenblatt M.S., Bennett W.P., Hollstein M., Harris C.C. (1994). Mutations in the p53 tumor suppressor gene: Clues to cancer etiology and molecular pathogenesis. Cancer Res..

[4] Resnick M.A., Inga A. (2003). Functional mutants of the sequence-specific transcription factor p53 and implications for master genes of diversity. Proc. Natl. Acad. Sci. USA.

[5] Ho T.L.F., Lee M.Y., Goh H.C., Ng G.Y.N., Lee J.J.H., Kannan S., Lim Y.T., Zhao T., Lim E.K.H., Phua C.Z.J. (2023). Domain-specific p53 mutants activate EGFR by distinct mechanisms exposing tissue-independent therapeutic vulnerabilities. Nat. Commun..

[6] Shi Y., Xie T., Wang B., Wang R., Cai Y., Yuan B., Gleber-Netto O.F., Tian X., Rodriguez-Rosario A.E., Osman A.A. (2022). Mutant p53 drives an immune cold tumor immune microenvironment in oral squamous cell carcinoma. Commun. Biol..

[7] Zhou X., Santos G.S., Zhan Y., Oliveira M.M.S., Rezaei S., Singh M., Peuget S., Westerberg L.S., Johnsen J.I., Selivanova G. (2022). Mutant p53 gain of function mediates cancer immune escape that is counteracted by APR-246. Br. J. Cancer.

[8] Lonetto G., Koifman G., Silberman A., Attery A., Solomon H., Levin-Zaidman S., Goldfinger N., Porat Z., Erez A., Rotter V. (2019). Mutant p53-dependent mitochondrial metabolic alterations in a mesenchymal stem cell-based model of progressive malignancy. Cell Death Differ..

[9] Schaafsma E., Takacs E.M., Kaur S., Cheng C., Kurokawa M. (2022). Predicting clinical outcomes of cancer patients with a p53 deficiency gene signature. Sci. Rep..

[10] Bian C., Li Z., Xu Y., Wang J., Xu L., Shen H. (2015). Clinical outcome and expression of mutant P53, P16, and Smad4 in lung adenocarcinoma: A prospective study. World J. Surg. Oncol..

[11] Choundhury S., Kolukula V., Preet A., Albanese C., Avantaggiati M.L. (2013). Dissecting the pathways that destabilize mutant p53: The proteasome or autophagy?. Cell Cycle.

[12] Kaida A., Iwakuma T. (2021). Regulation of p53 and Cancer Signaling by Heat Shock Protein 40/J-Domain Protein Family Members. Int. J. Mol. Sci..

[13] Wiech M., Olszewski M.B., Tracz-Gaszewska Z., Wawrzynow B., Zylicz M., Zylicz A. (2012). Molecular mechanism of mutant p53 stabilization: The role of HSP70 and MDM2. PLoS ONE.

[14] Cordani M., Butera G., Dando I., Torrens-Mas M., Butturini E., Pacchiana R., Oppici E., Cavallini C., Gasperini S., Tamassia N. (2018). Mutant p53 blocks SESN1/AMPK/PGC-1α/UCP2 axis increasing mitochondrial O2-· production in cancer cells. Br. J. Cancer.

[15] Dando I., Cordani M., Donadelli M. (2016). Mutant p53 and mTOR/PKM2 regulation in cancer cells. IUBMB Life.

[16] Schofield H.K., Zeller J., Espinoza C., Halbrook C.J., del Vecchio A., Magnuson B., Fabo T., Cali Daylan A.E., Kovalenko I., Lee H.J. (2018). Mutant p53R270H drives altered metabolism and increased invasion in pancreatic ductal adenocarcinoma. JCI Insight.

[17] Kollareddy M., Dimitrova E., Vallabhaneni K.C., Chan A., Le T., Chauhan K.M., Carrero Z.I., Ramakrishnan G., Watabe K., Haupt Y. (2015). Regulation of nucleotide metabolism by mutant p53 contributes to its gain-of-function activities. Nat. Commun..

[18] Tombari C., Zannini A., Bertolio R., Pedretti S., Audano M., Triboli L., Cancila V., Vacca D., Caputo M., Donzelli S. (2023). Mutant p53 sustains serine-glycine synthesis and essential amino acids intake promoting breast cancer growth. Nat. Commun..

[19] Ding D., Blee A.M., Zhang J., Pan Y., Becker N.A., Maher L.J., Jimenez R., Wang L., Huang H. (2023). Gain-of-function mutant p53 together with ERG proto-oncogene drive prostate cancer by beta-catenin activation and pyrimidine synthesis. Nat. Commun..

[20] Wang Z.H., Peng W.B., Zhang P., Yang X.P., Zhou Q. (2021). Lactate in the tumour microenvironment: From immune modulation to therapy. EBioMedicine.

[21] Zhang C., Liu J., Liang Y., Wu R., Zhao Y., Hong X., Lin M., Yu H., Liu L., Levine A.J. (2013). Tumour-associated mutant p53 drives the Warburg effect. Nat. Commun..

[22] Eriksson M., Ambroise G., Ouchida A.T., Lima Queiroz A., Smith D., Gimenez-Cassina A., Iwanicki M.P., Muller P.A., Norberg E., Vakifahmetoglu-Norberg H. (2017). Effect of Mutant p53 Proteins on Glycolysis and Mitochondrial Metabolism. Mol. Cell Biol..

[23] Chaudagar K., Hieromnimon H.M., Khurana R., Labadie B., Hirz T., Mei S., Hasan R., Shafran J., Kelley A., Apostolov E. (2023). Reversal of Lactate and PD-1–mediated Macrophage Immunosuppression Controls Growth of PTEN/p53-deficient Prostate Cancer. Clin. Cancer Res..

[24] Cordani M., Pacchiana R., Butera G., D’Orazi G., Scarpa A., Donadelli M. (2016). Mutant p53 proteins alter cancer cell secretome and tumour microenvironment: Involvement in cancer invasion and metastasis. Cancer Lett..

[25] Butera G., Brandi J., Cavallini C., Scarpa A., Lawlor R.T., Scupoli M.T., Marengo E., Cecconi D., Manfredi M., Donadelli M. (2020). The Mutant p53-Driven Secretome Has Oncogenic Functions in Pancreatic Ductal Adenocarcinoma Cells. Biomolecules.

[26] Capaci V., Bascetta L., Fantuz M., Beznoussenko G.V., Sommaggio R., Cancila V., Bisso A., Campaner E., Mironov A.A., Wiśniewski J.R. (2020). Mutant p53 induces Golgi tubulo-vesiculation driving a prometastatic secretome. Nat. Commun..

[27] Wright K., Ly T., Kriet M., Czirok A., Thomas S.M. (2023). Cancer-Associated Fibroblasts: Master Tumor Microenvironment Modifiers. Cancers.

[28] O’Loghlen A. (2018). Role for extracellular vesicles in the tumour microenvironment. Philos. Trans. R. Soc. Lond. B Biol. Sci..

[29] Novo D., Heath N., Mitchell L., Caligiuri G., MacFarlane A., Reijmer D., Charlton L., Knight J., Calka M., McGhee E. (2018). Mutant p53s generate pro-invasive niches by influencing exosome podocalyxin levels. Nat. Commun..

[30] Bhatta B., Luz I., Krueger C., Teo F.X., Lane D.P., Sabapathy K., Cooks T. (2021). Cancer Cells Shuttle Extracellular Vesicles Containing Oncogenic Mutant p53 Proteins to the Tumor Microenvironment. Cancers.

[31] Ju Q., Zhao L., Gao J., Zhou L., Xu Y., Sun Y., Zhao X. (2019). Mutant p53 increases exosome-mediated transfer of miR-21-3p and miR-769-3p to promote pulmonary metastasis. Chin. J. Cancer Res..

[32] Ma S., McGuire M.H., Mangala L.S., Lee S., Stur E., Hu W., Bayraktar E., Villar-Prados A., Ivan C., Wu S.Y. (2021). Gain-of-function p53 protein transferred via small extracellular vesicles promotes conversion of fibroblasts to a cancer-associated phenotype. Cell Rep..

[33] Dong X., Li C., Deng C., Liu J., Li D., Zhou T., Yang X., Liu Y., Guo Q., Feng Y. (2024). Regulated secretion of mutant p53 negatively affects T lymphocytes in the tumor microenvironment. Oncogene.

[34] Cooks T., Pateras I.S., Jenkins L.M., Patel K.M., Robles A.I., Morris J., Forshew T., Appella E., Gorgoulis V.G., Harris C.C. (2018). Mutant p53 cancers reprogram macrophages to tumor supporting macrophages via exosomal miR-1246. Nat. Commun..

[35] Ambrosini G., Cordani M., Zarrabi A., Alcon-Rodriguez S., Sainz R.M., Velasco G., Gonzalez-Menendez P., Dando I. (2024). Transcending frontiers in prostate cancer: The role of oncometabolites on epigenetic regulation, CSCs, and tumor microenvironment to identify new therapeutic strategies. Cell Commun. Signal..

[36] Fu Y., Yu J., Li F., Ge S. (2022). Oncometabolites drive tumorigenesis by enhancing protein acylation: From chromosomal remodelling to nonhistone modification. J. Exp. Clin. Cancer Res..

[37] Madrigal T., Ortega-Bernal D., Herrera L.A., González-De la Rosa C.H., Domínguez-Gómez G., Aréchaga-Ocampo E., Díaz-Chávez J. (2023). Mutant p53 Gain-of-Function Induces Migration and Invasion through Overexpression of miR-182-5p in Cancer Cells. Cells.

[38] Klemke L., Fehlau C.F., Winkler N., Toboll F., Singh S.K., Moll U.M., Schulz-Heddergott R. (2021). The Gain-of-Function p53 R248W Mutant Promotes Migration by STAT3 Deregulation in Human Pancreatic Cancer Cells. Front. Oncol..

[39] Romeo M.A., Gilardini Montani M.S., Benedetti R., Santarelli R., D’Orazi G., Cirone M. (2020). STAT3 and mutp53 Engage a Positive Feedback Loop Involving HSP90 and the Mevalonate Pathway. Front. Oncol..

[40] Lakoduk A.M., Roudot P., Mettlen M., Grossman H.M., Schmid S.L., Chen P.H. (2019). Mutant p53 amplifies a dynamin-1/APPL1 endosome feedback loop that regulates recycling and migration. J. Cell Biol..

[41] Mukherjee S., Maddalena M., Lü Y., Martinez S., Nataraj N.B., Noronha A., Sinha S., Teng K., Cohen-Kaplan V., Ziv T. (2022). Cross-talk between mutant p53 and p62/SQSTM1 augments cancer cell migration by promoting the degradation of cell adhesion proteins. Proc. Natl. Acad. Sci. USA.

[42] Pavlakis E., Neumann M., Merle N., Wieboldt R., Wanzel M., Ponath V., Pogge von Strandmann E., Elmshäuser S., Stiewe T. (2023). Mutant p53-ENTPD5 control of the calnexin/calreticulin cycle: A druggable target for inhibiting integrin-α5-driven metastasis. J. Exp. Clin. Cancer Res..

[43] Sun S., Chen H., Sun L., Wang M., Wu X., Xiao Z.X.J. (2020). Hotspot mutant p53-R273H inhibits KLF6 expression to promote cell migration and tumor metastasis. Cell Death Dis..

[44] Lv T., Lv H., Fei J., Xie Y., Lian D., Hu J., Tang L., Shi X., Wang J., Zhang S. (2020). p53-R273H promotes cancer cell migration via upregulation of neuraminidase-1. J. Cancer.

[45] Torrens-Mas M., Cordani M., Mullappilly N., Pacchiana R., Riganti C., Palmieri M., Pons D.G., Roca P., Oliver J., Donadelli M. (2020). Mutant p53 induces SIRT3/MnSOD axis to moderate ROS production in melanoma cells. Arch. Biochem. Biophys..

[46] Luo Q., Beaver J.M., Liu Y., Zhang Z. (2017). Dynamics of p53: A Master Decider of Cell Fate. Genes.

[47] Yi J., Luo J. (2010). SIRT1 and p53, effect on cancer, senescence and beyond. Biochim. Biophys. Acta.

[48] Tang Y., Zhao W., Chen Y., Zhao Y., Gu W. (2008). Acetylation is indispensable for p53 activation. Cell.

[49] Stanga S., Lanni C., Govoni S., Uberti D., D’Orazi G., Racchi M. (2010). Unfolded p53 in the pathogenesis of Alzheimer’s disease: Is HIPK2 the link?. Aging.

[50] Kupis W., Pałyga J., Tomal E., Niewiadomska E. (2016). The role of sirtuins in cellular homeostasis. J. Physiol. Biochem..

[51] Blander G., Guarente L. (2004). The Sir2 family of protein deacetylases. Annu. Rev. Biochem..

[52] Langley E., Pearson M., Faretta M., Bauer U.M., Frye R.A., Minucci S., Pelicci P.G., Kouzarides T. (2002). Human SIR2 deacetylates p53 and antagonizes PML/p53-induced cellular senescence. EMBO J..

[53] Puca R., Nardinocchi L., Starace G., Rechavi G., Sacchi A., Givol D., D’Orazi G. (2010). Nox1 is involved in p53 deacetylation and suppression of its transcriptional activity and apoptosis. Free Radic. Biol. Med..

[54] Aventaggiato M., Vernucci E., Barreca F., Russo M.A., Tafani M. (2021). Sirtuins’ control of autophagy and mitophagy in cancer. Pharmacol. Ther..

[55] Yamakuchi M., Ferlito M., Lowenstein C.J. (2008). miR-34a repression of SIRT1 regulates apoptosis. Proc. Natl. Acad. Sci. USA.

[56] Jeong J., Juhn K., Lee H., Kim S.H., Min B.H., Lee K.M., Cho M.H., Park G.H., Lee K.H. (2007). SIRT1 promotes DNA repair activity and deacetylation of Ku70. Exp. Mol. Med..

[57] Zhang Y.Y., Zhou L.M. (2012). Sirt3 inhibits hepatocellular carcinoma cell growth through reducing Mdm2-mediated p53 degradation. Biochem. Biophys. Res. Commun..

[58] Yin J.Y., Lu X.T., Hou M.L., Cao T., Tian Z. (2023). Sirtuin1-p53: A potential axis for cancer therapy. Biochem. Pharmacol..

[59] Ito A., Lai C.H., Zhao X., Saito S., Hamilton M.H., Appella E., Yao T.P. (2001). p300/CBP-mediated p53 acetylation is commonly induced by p53-activating agents and inhibited by MDM2. EMBO J..

[60] Perez R.E., Knights C.D., Sahu G., Catania J., Kolukula V.K., Stoler D., Graessmann A., Ogryzko V., Pishvaian M., Albanese C. (2010). Restoration of DNA-binding and growth-suppressive activity of mutant forms of p53 via a PCAF-mediated acetylation pathway. J. Cell Physiol..

[61] Yi Y.W., Kang H.J., Kim H.J., Kong Y., Brown M.L., Bae I. (2013). Targeting mutant p53 by a SIRT1 activator YK-3-237 inhibits the proliferation of triple-negative breast cancer cells. Oncotarget.

[62] Puca R., Nardinocchi L., Sacchi A., Rechavi G., Givol D., D’Orazi G. (2009). HIPK2 modulates p53 activity towards pro-apoptotic transcription. Mol. Cancer.

[63] Minamoto T., Buschmann T., Habelhah H., Matusevich E., Tahara H., Boerresen-Dale A.L., Harris C., Sidransky D., Ronai Z. (2001). Distinct pattern of p53 phosphorylation in human tumors. Oncogene.

[64] Rodriguez O.C., Choudhury S., Kolukula V., Vietsch E.E., Catania J., Preet A., Reynoso K., Bargonetti J., Wellstein A., Albanese C. (2012). Dietary downregulation of mutant p53 levels via glucose restriction: Mechanisms and implications for tumor therapy. Cell Cycle.

[65] Tang X., Li Y., Liu L., Guo R., Zhang P., Zhang Y., Zhang Y., Zhao J., Su J., Sun L. (2020). Sirtuin 3 induces apoptosis and necroptosis by regulating mutant p53 expression in small-cell lung cancer. Oncol. Rep..

[66] Tao R., Vassilopoulos A., Parisiadou L., Yan Y., Gius D. (2014). Regulation of MnSOD enzymatic activity by Sirt3 connects the mitochondrial acetylome signaling networks to aging and carcinogenesis. Antioxid. Redox Signal..

[67] Treviño-Saldaña N., García-Rivas G. (2017). Regulation of Sirtuin-Mediated Protein Deacetylation by Cardioprotective Phytochemicals. Oxid. Med. Cell Longev..

[68] Suenkel B., Valente S., Zwergel C., Weiss S., Di Bello E., Fioravanti R., Aventaggiato M., Amorim J.A., Garg N., Kumar S. (2022). Potent and Specific Activators for Mitochondrial Sirtuins Sirt3 and Sirt5. J. Med. Chem..

[69] Zwergel C., Aventaggiato M., Garbo S., Di Bello E., Fassari B., Noce B., Castiello C., Lambona C., Barreca F., Rotili D. (2023). Novel 1,4-Dihydropyridines as Specific Binders and Activators of SIRT3 Impair Cell Viability and Clonogenicity and Downregulate Hypoxia-Induced Targets in Cancer Cells. J. Med. Chem..

[70] Wang G., Wang J.J., To T.S.S., Zhao H.F., Wang J. (2015). Role of SIRT1-mediated mitochondrial and Akt pathways in glioblastoma cell death induced by Cotinus coggygria flavonoid nanoliposomes. Int. J. Nanomed..

[71] Wang G., Wang J.J., Wang Y.Z., Feng S., Jing G., Fu X.L. (2018). Myricetin nanoliposomes induced SIRT3-mediated glycolytic metabolism leading to glioblastoma cell death. Artif. Cells Nanomed. Biotechnol..

[72] Aventaggiato M., Barreca F., Sansone L., Pellegrini L., Russo M.A., Cordani M., Tafani M. (2022). Sirtuins and Hypoxia in EMT Control. Pharmaceuticals.

[73] Lukashchuk N., Vousden K.H. (2007). Ubiquitination and degradation of mutant p53. Mol.Cell Biol..

[74] Schulz-Heddergott R., Moll U.M. (2018). Gain-of-Function (GOF) Mutant p53 as Actionable Therapeutic Target. Cancers.

[75] Wang J., Liu W., Zhang L., Zhang J. (2023). Targeting mutant p53 stabilization for cancer therapy. Front. Pharmacol..

[76] do Patrocinio A.B., Rodrigues V., Guidi Magalhães L. (2022). P53: Stability from the Ubiquitin–Proteasome System and Specific 26S Proteasome Inhibitors. ACS Omega.

[77] Lee J.T., Gu W. (2010). The multiple levels of regulation by p53 ubiquitination. Cell Death Differ..

[78] Kocaturk N.M., Gozuacik D. (2018). Crosstalk between Mammalian Autophagy and the Ubiquitin-Proteasome System. Front. Cell Dev. Biol..

[79] Olive K.P., Tuveson D.A., Ruhe Z.C., Yin B., Willis N.A., Bronson R.T., Crowley D., Jacks T. (2004). Mutant p53 Gain of Function in Two Mouse Models of Li-Fraumeni Syndrome. Cell.

[80] Lang G.A., Iwakuma T., Suh Y.A., Liu G., Rao V.A., Parant J.M., Valentin-Vega Y.A., Terzian T., Caldwell L.C., Strong L.C. (2004). Gain of Function of a p53 Hot Spot Mutation in a Mouse Model of Li-Fraumeni Syndrome. Cell.

[81] Terzian T., Suh Y.A., Iwakuma T., Post S.M., Neumann M., Lang G.A., Van Pelt C.S., Lozano G. (2008). The inherent instability of mutant p53 is alleviated by *Mdm2* or *p16 ^INK4a^* loss. Genes. Dev..

[82] Prives C., White E. (2008). Does control of mutant p53 by Mdm2 complicate cancer therapy?. Genes Dev..

[83] Zheng T., Wang J., Zhao Y., Zhang C., Lin M., Wang X., Yu H., Liu L., Feng Z., Hu W. (2013). Spliced MDM2 isoforms promote mutant p53 accumulation and gain-of-function in tumorigenesis. Nat. Commun..

[84] Zhang Y., Xiong S., Li Q., Hu S., Tashakori M., Van Pelt C., You M.J., Pageon L., Lozano G. (2014). Tissue-specific and age-dependent effects of global Mdm2 loss. J. Pathol..

[85] Whitesell L., Lindquist S.L. (2005). HSP90 and the chaperoning of cancer. Nat. Rev. Cancer.

[86] Peng Y., Chen L., Li C., Lu W., Chen J. (2001). Inhibition of MDM2 by hsp90 contributes to mutant p53 stabilization. J. Biol. Chem..

[87] Parrales A., Ranjan A., Iyer S.V., Padhye S., Weir S.J., Roy A., Iwakuma T. (2016). DNAJA1 controls the fate of misfolded mutant p53 through the mevalonate pathway. Nat. Cell Biol..

[88] Romeo M.A., Gilardini Montani M.S., Arena A., Benedetti R., D’Orazi G., Cirone M. (2022). c-Myc Sustains Pancreatic Cancer Cell Survival and mutp53 Stability through the Mevalonate Pathway. Biomedicines.

[89] Liu J., Zhang C., Xu D., Zhang T., Chang C.Y., Wang J., Liu J., Zhang L., Haffty B.G., Zong W.X. (2023). The ubiquitin ligase TRIM21 regulates mutant p53 accumulation and gain of function in cancer. J. Clin. Investig..

[90] Shi Y., Shen H.M., Gopalakrishnan V., Gordon N. (2021). Epigenetic Regulation of Autophagy Beyond the Cytoplasm: A Review. Front. Cell Dev. Biol..

[91] Galluzzi L., Baehrecke E.H., Ballabio A., Boya P., Bravo-San Pedro J.M., Cecconi F., Choi A.M., Chu C.T., Codogno P., Colombo M.I. (2017). Molecular definitions of autophagy and related processes. EMBO J..

[92] Oku M., Sakai Y. (2018). Three Distinct Types of Microautophagy Based on Membrane Dynamics and Molecular Machineries. BioEssays..

[93] Cuervo A.M., Dice J.F. (1996). A Receptor for the Selective Uptake and Degradation of Proteins by Lysosomes. Science.

[94] Kaushik S., Cuervo A.M. (2008). Chaperone-Mediated Autophagy. https://pubmed.ncbi.nlm.nih.gov/18425454/.

[95] LaGory E.L., Giaccia A.J. (2013). A low-carb diet kills tumor cells with a mutant p53 tumor suppressor gene. Cell Cycle.

[96] Garufi A., Pistritto G., Cirone M., D’Orazi G. (2016). Reactivation of mutant p53 by capsaicin, the major constituent of peppers. J. Exp. Clin. Cancer Res..

[97] Garufi A., Federici G., Gilardini Montani M.S., Crispini A., Cirone M., D’Orazi G. (2020). Interplay between Endoplasmic Reticulum (ER) Stress and Autophagy Induces Mutant p53H273 Degradation. Biomolecules.

[98] Garufi A., Pucci D., D’Orazi V., Cirone M., Bossi G., Avantaggiati M.L., D’Orazi G. (2014). Degradation of mutant p53H175 protein by Zn(II) through autophagy. Cell Death Dis..

[99] Foggetti G., Ottaggio L., Russo D., Monti P., Degan P., Fronza G., Menichini P. (2017). Gambogic acid counteracts mutant p53 stability by inducing autophagy. Biochim. Et. Biophys. Acta (BBA) Mol. Cell Res..

[100] Baldari S., Ubertini V., Garufi A., D’Orazi G., Bossi G. (2015). Targeting MKK3 as a novel anticancer strategy: Molecular mechanisms and therapeutical implications. Cell Death Dis..

[101] Aggarwal M., Saxena R., Sinclair E., Fu Y., Jacobs A., Dyba M., Wang X., Cruz I., Berry D., Kallakury B. (2016). Reactivation of mutant p53 by a dietary-related compound phenethyl isothiocyanate inhibits tumor growth. Cell Death Differ..

[102] Mrakovcic M., Kleinheinz J., Fröhlich L. (2017). Histone Deacetylase Inhibitor-Induced Autophagy in Tumor Cells: Implications for p53. Int. J. Mol. Sci..

[103] Garufi A., Trisciuoglio D., Porru M., Leonetti C., Stoppacciaro A., D’Orazi V., Avantaggiati M., Crispini A., Pucci D., D’Orazi G. (2013). A fluorescent curcumin-based Zn(II)-complex reactivates mutant (R175H and R273H) p53 in cancer cells. J. Exp. Clin. Cancer Res..

[104] Garufi A., Pistritto G., Baldari S., Toietta G., Cirone M., D’Orazi G. (2017). p53-Dependent PUMA to DRAM antagonistic interplay as a key molecular switch in cell-fate decision in normal/high glucose conditions. J. Exp. Clin. Cancer Res..

[105] Cuervo A.M., Knecht E., Terlecky S.R., Dice J.F. (1995). Activation of a selective pathway of lysosomal proteolysis in rat liver by prolonged starvation. Am. J. Physiol. Cell Physiol..

[106] Vakifahmetoglu-Norberg H., Kim M., Xia H.G., Iwanicki M.P., Ofengeim D., Coloff J.L., Pan L., Ince T.A., Kroemer G., Brugge J.S. (2013). Chaperone-mediated autophagy degrades mutant p53. Genes. Dev..

[107] Wu R., Galan-Acosta L., Norberg E. (2015). Glucose metabolism provide distinct prosurvival benefits to non-small cell lung carcinomas. Biochem. Biophys. Res. Commun..

[108] Benedetti R., Romeo M.A., Arena A., Gilardini Montani M.S., D’Orazi G., Cirone M. (2024). ATF6 supports lysosomal function in tumor cells to enable ER stress-activated macroautophagy and CMA: Impact on mutant TP53 expression. Autophagy.

[109] Cuervo A.M., Dice J.F. (2000). Age-related Decline in Chaperone-mediated Autophagy. J. Biol. Chem..

[110] Pajares M., Rojo A.I., Arias E., Díaz-Carretero A., Cuervo A.M., Cuadrado A. (2018). Transcription factor NFE2L2/NRF2 modulates chaperone-mediated autophagy through the regulation of LAMP2A. Autophagy.

[111] Lisek K., Campaner E., Ciani Y., Walerych D., Del Sal G. (2018). Mutant p53 tunes the NRF2-dependent antioxidant response to support survival of cancer cells. Oncotarget.

[112] Tekirdag K., Cuervo A.M. (2018). Chaperone-mediated autophagy and endosomal microautophagy: Jointed by a chaperone. J. Biol. Chem..

[113] Kwon J., Kim J., Kim K.I. (2023). Crosstalk between endoplasmic reticulum stress response and autophagy in human diseases. Anim. Cells Syst..

[114] Garufi A., Pistritto G., D’Orazi V., Cirone M., D’Orazi G. (2022). The Impact of NRF2 Inhibition on Drug-Induced Colon Cancer Cell Death and p53 Activity: A Pilot Study. Biomolecules.

[115] Russell R.C., Guan K. (2022). The multifaceted role of autophagy in cancer. EMBO J..

[116] Galluzzi L., Pietrocola F., Bravo-San Pedro J.M., Amaravadi R.K., Baehrecke E.H., Cecconi F., Codogno P., Debnath J., Gewirtz D.A., Karantza V. (2015). Autophagy in malignant transformation and cancer progression. EMBO J..

[117] Behrooz A.B., Cordani M., Donadelli M., Ghavami S. (2023). Metastatic outgrowth via the two-way interplay of autophagy and metabolism. Biochim. Biophys. Acta Mol. Basis Dis..

[118] Mowers E.E., Sharifi M.N., Macleod K.F. (2018). Functions of autophagy in the tumor microenvironment and cancer metastasis. FEBS J..

[119] Di Leo L., Bodemeyer V., Bosisio F.M., Claps G., Carretta M., Rizza S., Faienza F., Frias A., Khan S., Bordi M. (2021). Loss of Ambra1 promotes melanoma growth and invasion. Nat. Commun..

[120] Cordani M., Oppici E., Dando I., Butturini E., Dalla Pozza E., Nadal-Serrano M., Oliver J., Roca P., Mariotto S., Cellini B. (2016). Mutant p53 proteins counteract autophagic mechanism sensitizing cancer cells to mTOR inhibition. Mol. Oncol..

[121] Zhou G., Wang J., Zhao M., Xie T.X., Tanaka N., Sano D., Patel A.A., Ward A.M., Sandulache V.C., Jasser S.A. (2014). Gain-of-Function Mutant p53 Promotes Cell Growth and Cancer Cell Metabolism via Inhibition of AMPK Activation. Mol. Cell.

[122] Morselli E., Tasdemir E., Maiuri M.C., Galluzzi L., Kepp O., Criollo A., Vicencio J.M., Soussi T., Kroemer G. (2008). Mutant p53 protein localized in the cytoplasm inhibits autophagy. Cell Cycle.

[123] Duffy M.J., Crown J. (2021). Drugging ‘undruggable’ genes for cancer treatment: Are we making progress?. Int. J. Cancer.

[124] Hassin O., Oren M. (2023). Drugging p53 in cancer: One protein, many targets. Nat. Rev. Drug Discov..

[125] Hu C., Yang J., Qi Z., Wu H., Wang B., Zou F., Mei H., Liu J., Wang W., Liu Q. (2022). Heat shock proteins: Biological functions, pathological roles, and therapeutic opportunities. MedComm.

[126] Boysen M., Kityk R., Mayer M.P. (2019). Hsp70- and Hsp90-Mediated Regulation of the Conformation of p53 DNA Binding Domain and p53 Cancer Variants. Mol. Cell.

[127] Helmbrecht K., Zeise E., Rensing L. (2000). Chaperones in cell cycle regulation and mitogenic signal transduction: A review. Cell Prolif..

[128] Blagosklonny M.V., Toretsky J., Bohen S., Neckers L. (1996). Mutant conformation of p53 translated in vitro or in vivo requires functional HSP90. Proc. Natl. Acad. Sci. USA.

[129] Muller P., Hrstka R., Coomber D., Lane D.P., Vojtesek B. (2008). Chaperone-dependent stabilization and degradation of p53 mutants. Oncogene.

[130] Li D., Yallowitz A., Ozog L., Marchenko N. (2014). A gain-of-function mutant p53-HSF1 feed forward circuit governs adaptation of cancer cells to proteotoxic stress. Cell Death Dis..

[131] Zylicz M., King F.W., Wawrzynow A. (2001). Hsp70 interactions with the p53 tumour suppressor protein. EMBO J..

[132] Garufi A., Baldari S., Pettinari R., Gilardini Montani M.S., D’Orazi V., Pistritto G., Crispini A., Giorno E., Toietta G., Marchetti F. (2020). A ruthenium(II)-curcumin compound modulates NRF2 expression balancing the cancer cell death/survival outcome according to p53 status. J. Exp. Clin. Cancer Res..

[133] Gilardini Montani M.S., Cecere N., Granato M., Romeo M.A., Falcinelli L., Ciciarelli U., D’Orazi G., Faggioni A. (2019). Cirone M Mutant p53, Stabilized by Its Interplay with HSP90, Activates a Positive Feed-Back Loop between NRF2 and p62 that Induces Chemo-Resistance to Apigenin in Pancreatic Cancer Cells. Cancers.

[134] Romeo M.A., Gilardini Montani M.S., Benedetti R., Arena A., D’Orazi G., Cirone M. (2022). VPA and TSA Interrupt the Interplay between mutp53 and HSP70, Leading to CHK1 and RAD51 Down-Regulation and Sensitizing Pancreatic Cancer Cells to AZD2461 PARP Inhibitor. Int. J. Mol. Sci..

[135] Gomes S., Bosco B., Loureiro J.B., Ramos H., Raimundo L., Soares J., Nazareth N., Barcherini V., Domingues L., Oliveira C. (2019). SLMP53-2 Restores Wild-Type-Like Function to Mutant p53 through Hsp70: Promising Activity in Hepatocellular Carcinoma. Cancers.

[136] Bykov V.J.N., Eriksson S.E., Bianchi J., Wiman K.G. (2018). Targeting mutant p53 for efficient cancer therapy. Nat. Rev. Cancer.

[137] King F.W., Wawrzynow A., Höhfeld J., Zylicz M. (2001). Co-chaperones Bag-1, Hop and Hsp40 regulate Hsc70 and Hsp90 interactions with wild-type or mutant p53. EMBO J..

[138] Esser C., Scheffner M., Höhfeld J. (2005). The Chaperone-associated Ubiquitin Ligase CHIP Is Able to Target p53 for Proteasomal Degradation. J. Biol. Chem..

[139] Quintana-Gallardo L., Martín-Benito J., Marcilla M., Espadas G., Sabidó E., Valpuesta J.M. (2019). The cochaperone CHIP marks Hsp70- and Hsp90-bound substrates for degradation through a very flexible mechanism. Sci. Rep..

[140] Hiraki M., Hwang S.Y., Cao S., Ramadhar T.R., Byun S., Yoon K.W., Lee J.H., Chu K., Gurkar A.U., Kolev V. (2015). Small-Molecule Reactivation of Mutant p53 to Wild-Type-like p53 through the p53-Hsp40 Regulatory Axis. Chem. Biol..

[141] Nishikawa S., Kaida A., Parrales A., Ranjan A., Alalem M., Ren H., Schoenen F.J., Johnson D.K., Iwakuma T. (2022). DNAJA1- and conformational mutant p53-dependent inhibition of cancer cell migration by a novel compound identified through a virtual screen. Cell Death Discov..

[142] Choi S.K., Kam H., Kim K.Y., Park S.I., Lee Y.S. (2019). Targeting Heat Shock Protein 27 in Cancer: A Druggable Target for Cancer Treatment?. Cancers.

[143] O’Callaghan-Sunol C., Gabai V.L., Sherman M.Y. (2007). Hsp27 Modulates p53 Signaling and Suppresses Cellular Senescence. Cancer Res..

[144] Kanagasabai R., Krishnamurthy K., Druhan L.J., Ilangovan G. (2011). Forced Expression of Heat Shock Protein 27 (Hsp27) Reverses P-Glycoprotein (ABCB1)-mediated Drug Efflux and MDR1 Gene Expression in Adriamycin-resistant Human Breast Cancer Cells. J. Biol. Chem..

[145] Nitika Porter C.M., Truman A.W., Truttmann M.C. (2020). Post-translational modifications of Hsp70 family proteins: Expanding the chaperone code. J. Biol. Chem..

[146] Backe S.J., Sager R.A., Woodford M.R., Makedon A.M., Mollapour M. (2020). Post-translational modifications of Hsp90 and translating the chaperone code. J. Biol. Chem..

[147] Lim S., Kim D.G., Kim S. (2019). ERK-dependent phosphorylation of the linker and substrate-binding domain of HSP70 increases folding activity and cell proliferation. Exp. Mol. Med..

[148] Muller P., Ruckova E., Halada P., Coates P.J., Hrstka R., Lane D.P., Vojtesek B. (2013). C-terminal phosphorylation of Hsp70 and Hsp90 regulates alternate binding to co-chaperones CHIP and HOP to determine cellular protein folding/degradation balances. Oncogene.

[149] Barra J., Cerda-Infante J., Sandoval L., Gajardo-Meneses P., Henriquez J.F., Labarca M., Metz C., Venegas J., Retamal C., Oyanadel C. (2021). D-Propranolol Impairs EGFR Trafficking and Destabilizes Mutant p53 Counteracting AKT Signaling and Tumor Malignancy. Cancers.

[150] Seo J.H., Park J.H., Lee E.J., Vo T.T.L., Choi H., Kim J.Y., Jang J.K., Wee H.J., Lee H.S., Jang S.H. (2016). ARD1-mediated Hsp70 acetylation balances stress-induced protein refolding and degradation. Nat. Commun..

[151] Kovacs J.J., Murphy P.J.M., Gaillard S., Zhao X., Wu J.T., Nicchitta C.V., Yoshida M., Toft D.O., Pratt W.B., Yao T.P. (2005). HDAC6 regulates Hsp90 acetylation and chaperone-dependent activation of glucocorticoid receptor. Mol. Cell.

[152] Li D., Marchenko N.D., Moll U.M. (2011). SAHA shows preferential cytotoxicity in mutant p53 cancer cells by destabilizing mutant p53 through inhibition of the HDAC6-Hsp90 chaperone axis. Cell Death Differ..

[153] Wang Y., Wang S.Y., Zhang X.H., Zhao M., Hou C.M., Xu Y.J., Du Z.Y., Yu X.D. (2007). FK228 inhibits Hsp90 chaperone function in K562 cells via hyperacetylation of Hsp70. Biochem. Biophys. Res. Commun..

[154] Orthwein A., Zahn A., Methot S.P., Godin D., Conticello S.G., Terada K., Di Noia J.M. (2012). Optimal functional levels of activation-induced deaminase specifically require the Hsp40 DnaJa1. EMBO J..

[155] Jakobsson M.E., Moen A., Bousset L., Egge-Jacobsen W., Kernstock S., Melki R., Falnes P.Ø. (2013). Identification and Characterization of a Novel Human Methyltransferase Modulating Hsp70 Protein Function through Lysine Methylation. J. Biol. Chem..

[156] Hamamoto R., Toyokawa G., Nakakido M., Ueda K., Nakamura Y. (2014). SMYD2-dependent HSP90 methylation promotes cancer cell proliferation by regulating the chaperone complex formation. Cancer Lett..

[157] Ban H., Han T.S., Hur K., Cho H.S. (2019). Epigenetic Alterations of Heat Shock Proteins (HSPs) in Cancer. Int. J. Mol. Sci..

[158] Coban N., Varol N. (2019). The effect of heat shock protein 90 inhibitors on histone 4 lysine 20 methylation in bladder cancer. EXCLI J..

[159] Tan Y.S., Mhoumadi Y., Verma C.S. (2019). Roles of computational modelling in understanding p53 structure, biology, and its therapeutic targeting. J. Mol. Cell Biol..

